# Supplementary material for: Agmatine Mitigates Inflammation-Related Oxidative Stress in BV-2 Cells by Inducing a Pre-Adaptive Response
Source: Int J Mol Sci. 2022 Mar 24;23(7):3561. doi: 10.3390/ijms23073561 (PMC8998340; doi:10.3390/ijms23073561)
Supplement: Supplementary file 1 [file ijms-23-03561-s001.zip › ijms-1624277-supplementary.pdf]

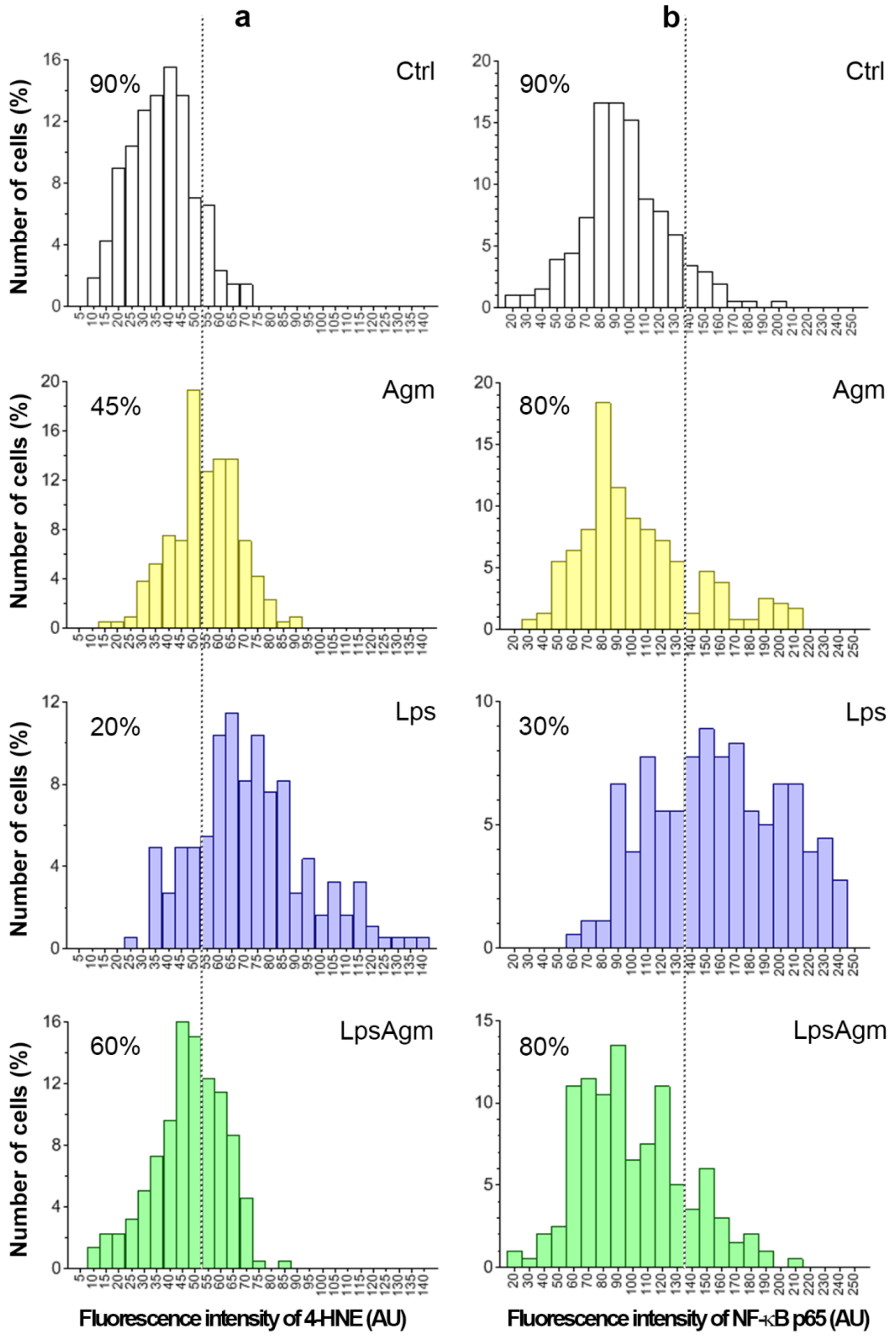

Figure S1. The percentage distribution of cellular 4-HNE and nuclear NF-κB p65 fluorescence intensity. (**a**) The percentage of cells within a specific range of 4-HNE fluorescence intensity grouped into 5 arbitrary units (AU) steps (the center of the first step is set at 5 AU, and the center of the last step is set at 140 AU). (**b**) The percentage number of cells showing nuclear NF-κB p65 fluorescence is grouped into 10 AU steps (the center of the first step is set at 20 AU, and the center of the last step is set at 250 AU). Vertical dashed lines on **a** and **b** represent the range limit containing 90% of control non-stimulateded BV-2 cells.
